# Crystallographic Study of Novel Transthyretin Ligands Exhibiting Negative-Cooperativity between Two Thyroxine Binding Sites

**DOI:** 10.1371/journal.pone.0043522

**Published:** 2012-09-04

**Authors:** Divya Tomar, Tarique Khan, Rajiv Ranjan Singh, Satyendra Mishra, Sarika Gupta, Avadhesha Surolia, Dinakar M. Salunke

**Affiliations:** 1 Structural Biology Unit, National Institute of Immunology, New Delhi, India; 2 Molecular Sciences Lab, National Institute of Immunology, New Delhi, India; 3 Centre for Molecular Medicine, National Institute of Immunology, New Delhi, India; 4 Molecular Biophysics Unit, Indian Institute of Sciences, Bangalore, India; 5 Regional Centre for Biotechnology, Gurgaon, India; Aligarh Muslim University, India

## Abstract

**Background:**

Transthyretin (TTR) is a homotetrameric serum and cerebrospinal fluid protein that transports thyroxine (T4) and retinol by binding to retinol binding protein. Rate-limiting tetramer dissociation and rapid monomer misfolding and disassembly of TTR lead to amyloid fibril formation in different tissues causing various amyloid diseases. Based on the current understanding of the pathogenesis of TTR amyloidosis, it is considered that the inhibition of amyloid fibril formation by stabilization of TTR in native tetrameric form is a viable approach for the treatment of TTR amyloidosis.

**Methodology and Principal Findings:**

We have examined interactions of the wtTTR with a series of compounds containing various substitutions at biphenyl ether skeleton and a novel compound, previously evaluated for binding and inhibiting tetramer dissociation, by x-ray crystallographic approach. High resolution crystal structures of five ligands in complex with wtTTR provided snapshots of negatively cooperative binding of ligands in two T4 binding sites besides characterizing their binding orientations, conformations, and interactions with binding site residues. In all complexes, the ligand has better fit and more potent interactions in first T4 site i.e. (AC site) than the second T4 site (BD site). Together, these results suggest that AC site is a preferred ligand binding site and retention of ordered water molecules between the dimer interfaces further stabilizes the tetramer by bridging a hydrogen bond interaction between Ser117 and its symmetric copy.

**Conclusion:**

Novel biphenyl ether based compounds exhibit negative-cooperativity while binding to two T4 sites which suggests that binding of only single ligand molecule is sufficient to inhibit the TTR tetramer dissociation.

## Introduction

Transthyretin (TTR) is a major protein in extracellular fluids which binds to thyroxine in two forms T_3_ (3,3′,5′-triiodothyronine) and T4 (3,5,3′,5′-tetraiodothyronine). While, in cerebrospinal fluid (CSF) TTR is the main thyroxine (T4) transport system, in plasma, TTR acts as a backup system to T4 binding globulins. Transthyretin also binds retinol-binding protein (RBP), in serum and CSF. It was first identified in human CSF and later in human serum. It is the only thyroxine-binding protein that is synthesized in the cells of the blood–CSF barrier (choroid plexus); however, its major site of synthesis is the liver. Transthyretin has been recognized as one of the most interesting proteins identified until date, because of its multi-functionality. Besides distributing thyroid hormones (THs) in blood, it indirectly transports vitamin A via binding to RBP. In addition, proteolytic activity of transthyretin has recently been discovered [1], adding to its importance in the central nervous system.

Transthyretin exists *in vivo* mainly as a tetramer of four identical subunits and only a small amount of the monomer [2]. Each subunit consists of 127 amino acid residues, which are largely arranged into β-sheet structure, which is believed to contribute to the extraordinary stability of the molecule [3]. X-ray studies of TTR show two funnel-shaped binding sites for T4, each defined by a dimer-dimer interface. In the quaternary structure, the TTR monomers are arranged into dimer-of-dimers configuration and form a β-sheet rich, homotetrameric structure [4], [5]. Under certain conditions, TTR can unfold and aggregate into amyloid fibrils [6], [7]. Several neurodegenerative and amyloid diseases such as familial amyloid cardiomyopathy (FAC), familial amyloid polyneuropathy (FAP) and senile systemic amyloidosis (SSA), are putatively caused by the formation of insoluble protein fibrils, which accumulate in human tissue and interfere with normal organ function [8]. These diseases are generally progressive and fatal, resulting in the death of the patient within 10–20 years. The underlying molecular mechanism which leads to the destabilization of a protein’s native conformation is still unclear.

The stabilization of TTR tetramer by thyroxine analogs, which bind to the two T4 binding pockets, has been the most promising strategy for stalling amyloidogenic potential of TTR [9]. This binding is known to stabilize TTR’s normally folded state, thus preventing the conformational changes required for amyloidogenicity [10], [11]. It has been observed that the association constants for the first and the second T4 molecule differ by a factor of about 100 [12], [13]. This difference in association constant values has been attributed to the negative cooperativity effect [12]. The ligands used in this study also have a drastic difference in the *K_d1_* and *K_d2_* values as determined by isothermal titration calorimetry in the previous study by Gupta *et al* [14]. Further it has also been shown that binding of single molecule is sufficient for stalling the process of TTR amyloidosis [15]. Here we present the crystal structures of wtTTR in complex with four biphenyl ether derivatives containing different functional groups and with a novel compound which was identified from a chemical library [14]. The derivatives of biphenyl ether were designed based on the triclosan skeleton, which has also been shown to stabilize TTR tetramer [16]. Efficacy of these compounds in stabilizing the tetramer against increasingly harsh desolvation conditions has previously been reported [14]. The crystallographic data presented here illustrates the structural basis of negative cooperativity between the two ligands and role of various substituted groups on biaryl skeleton in imparting stability to TTR tetramer in amyloidogenic conditions.

## Results

### Crystal Structures

**Figure 1:**
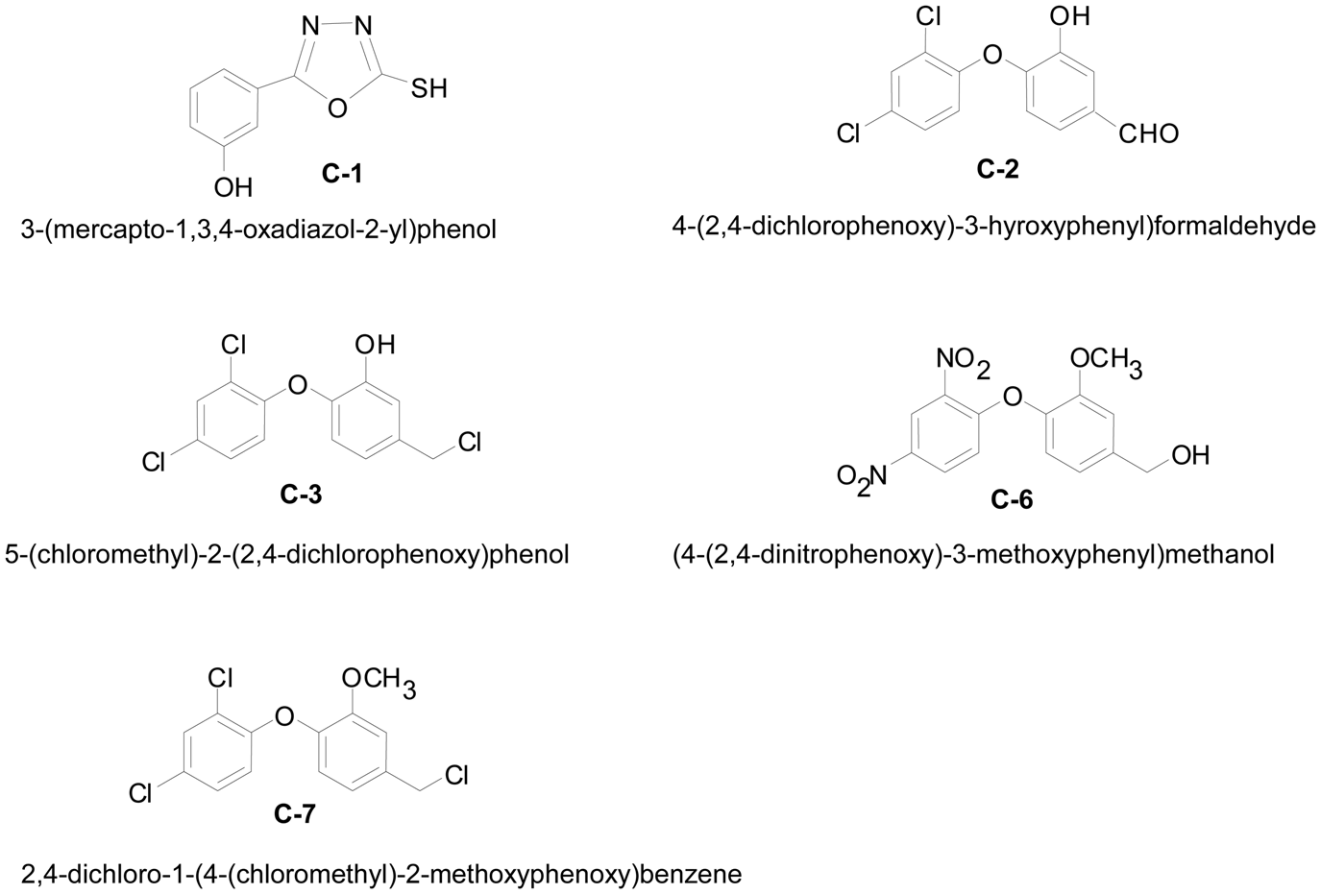
Representation of bi-dimensional chemical structures of the ligands used in this study. C1{3-(5-mercapto-1,3,4oxadiazol-2-yl)-phenol}, C2 {4-(2,4-dichlorophenoxy)-3-hydroxyphenyl)formaldehyde}, C3 {5-(chloromethyl)-2-(2,4-dichlorophenoxy)phenol}, C6 {(4-(2,4-dinitrophenoxy)-3-methoxyphenyl)methanol}, and C7 {2,4-dichloro-1-(4 (chloromethyl)-2-methoxyphenoxy)benzene}.

The wtTTR crystal structures with compounds C1{3-(5-mercapto-1,3,4 oxadiazol-2-yl)-phenol}, C2 {4-(2,4-dichlorophenoxy)-3-hydroxyphenyl)formaldehyde}, C3 {5-(chloromethyl)-2-(2,4-dichlorophenoxy)phenol}, C6 {(4-(2,4-dinitrophenoxy)-3-methoxyphenyl)methanol}, and C7 {2,4-dichloro-1-(4(chloromethyl)-2-methoxyphenoxy)benzene} (Figure 1) were obtained by soaking of the native TTR crystals into the crystallization media containing saturating amounts of the respective compounds. The inhibitory potential of compounds C1, C2, C3, C6, and C7 were previously evaluated and they were designated serial numbers 11, 2, 3, 19, and 5, respectively [14]. The crystal structures of all complexes of wtTTR were determined by molecular replacement using the coordinates of native TTR (pdb code: 1DVQ). Data collection and refinement statistics are given in Table 1. Amino acids 10–124 were well-defined in the electron density maps of all the complexes. The unambiguous electron densities in difference maps contoured at 3*σ* in all the five structures allowed precise placement of the ligands. After final refinement and water search, no other large and continuous electron density was found, ruling out the possibility of ligand binding to other TTR regions in the crystal.

**Table 1:** Data collection and refinement statistics.

	wtTTR	wtTTR-C1	wtTTR-C2	wtTTR-C3	wtTTR-C6	wtTTR-C7
**Data collection**						
Space group	P2_1_2_1_2	P2_1_2_1_2	P2_1_2_1_2	P2_1_2_1_2	P2_1_2_1_2	P2_1_2_1_2
Cell dimensions						
a, b, c (Å)	85.9, 42.5, 64.4	85.7, 42.6, 63.9	85.6, 42.4, 63.8	85.7, 42.8, 64.5	85.8, 42.8, 64.6	85.5, 42.5, 64.5
α = β = γ (°)	90.0	90.0	90.0	90.0	90.0	90.0
Resolution (Å)	35.7−1.7	35.6−1.7	35.5−1.86	35.7−1.8	35.7−1.7	42.7−1.8
R_merge_	0.028	0.052	0.066	0.049	0.069	0.075
(I)/(σ I)	19.0 (4.6)	17.0 (3.0)	12.5 (2.9)	22.3 (3.1)	10.7 (3.9)	17.8 (5.6)
Completeness (%)	99.3 (98.0)	99.7 (98.8)	99.8 (99.5)	99.7 (97.8)	99.9 (100)	99.5 (96.5)
Multiplicity	4.8	6.2	5.3	7.2	5.1	8.5
**Refinement**						
Resolution (Å)	35.7−1.7	63.9−1.7	63.8−1.86	35.7−1.8	50−1.7	64.0−1.8
No. of reflections	23,804	23,760	19,121	18,201	23,681	19,864
R_work/_R_free_ (%)	21.0/24.9	20.4/24.1	20.5/25.3	20.4/24.6	21.8/25.7	18.7/23.5
**No. of atoms**						
Water	112	134	127	114	170	180
Protein	1779	1779	1779	1779	1779	1779
Ligand	–	26	36	36	46	38
**B-factors (Å^2^)**						
Water	29.5	33.7	38.3	35.6	35.7	35.7
Protein	17.7	22.6	25.0	26.0	24.3	24.3
Ligand	–	38.0	49.3	47.6	51.3	51.3
**RMSDs**						
Bond lengths (Å)	0.02	0.02	0.02	0.02	0.02	0.02
Bond angles (°)	2.1	2.2	2.0	1.9	2.1	2.0
**Ramachandran statistics (MolProbity ** [**30**] **)**						
Favored (%)	99.1	98.3	100	98.0	98.6	98.6
Allowed (%)	0.9	1.7	0	2.0	1.4	1.4

Data for the outermost shell are given in parentheses.

### Overall Structure of TTR

**Figure 2:**
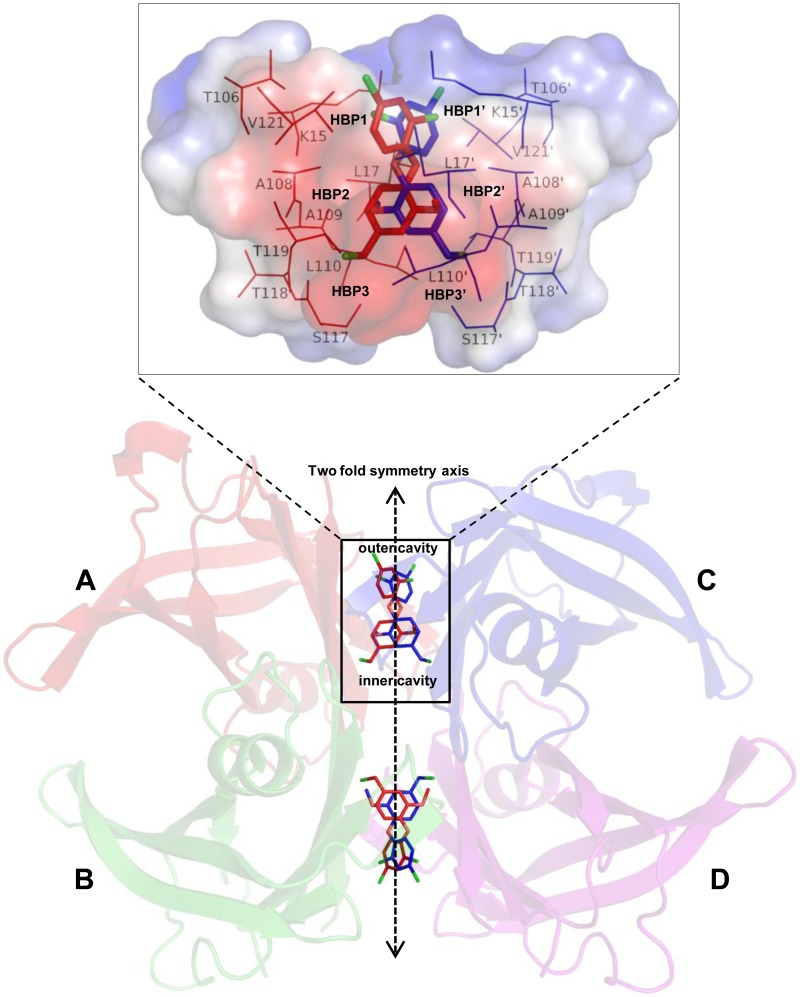
Prototype structure of the wtTTR-ligand complex. Ribbon diagram of tetrameric TTR with C7 is displayed. The two molecules of C7, binding into the T4-binding channels of TTR, are shown as a sticks. Because of the two fold symmetry axis along the binding channel (pointing from top to bottom in the paper plane) a second symmetry-related binding mode is present for both molecules. For clarity, ribbon diagram is made transparent, Ligand C7 is shown in red color and its symmetric copy is shown in blue color. Non crystallographic (AB) and crystallographic (CD) dimers are shown sideways to assemble the tetrameric molecule. Enlarged view of the T4 binding site constituted by A and C monomer is displayed as transparent surface with charge distribution (red, hydrophobic; blue, charged) and amino acid residues forming the HBP1-HBP1’, HBP2-HBP2’ and HBP3-HBP3’ are shown as thin sticks.

As in all previously reported structures, TTR was crystallized in P2_1_2_1_2 space group with two monomers in an asymmetric unit which are not related by non crystallographic symmetry. Each dimer generates a tetramer from crystallographic symmetry-related dimer of itself as shown in Figure 2. Therefore, tetrameric TTR consists of two dimers: A–B and C–D (Figure 2). Each monomer of TTR consists of 127 residues forming 8 β-strands and 1 α-helix, which fold into the two layers of a β-sheet, producing a β-sandwich structure. Last 3 carboxy terminal residues were disordered in all complexes. As shown in Figure 2, the quaternary structure of the TTR contains two T4 binding sites constituted by the interface of monomers A–C and B–D, respectively. Each T4 binding site contains three pairs of halogen binding pockets (HBP1-HBP1′, HBP2-HBP2′ and HBP3-HBP3′), wherein the iodine atoms of T4 reside. One pair is located in each of the smaller inner and larger outer binding cavities, while the third is found at the interface between the two cavities (Figure 2). As shown in the zoomed view of the ligand binding site in Figure 2, the inner binding cavity comprises of HBP3 and its twofold symmetry related counterpart HBP3′, formed by the side chains of Ser117, Leu110, Thr119 and Ala108 and their twofold symmetric counterparts. Similarly, the outer binding site comprises of HBP1 and its twofold symmetry related HBP1′ formed by residues Lys15, Leu17, Thr106, and Val121. Central pocket is formed by HBP2 and HBP2′, comprising of residues Leu17, Ala108, Ala109, and Leu110 as well as methylene carbons of Lys15 side chain and the twofold symmetric counterpart of each of these residues. In the final models, each ligand was found to lay exactly at a twofold symmetry along Z axis in the T4 binding pocket and hence generates exactly superimposable symmetric copy of itself (colored red and blue in Figure 2). Therefore, when one ligand copy interacts with HBP3/HBP2/HBP1, its symmetric counterpart interacts with HBP3′/HBP2′/HBP1′. A prototype depiction to clearly mark the outer and inner binding cavities along with 3 HBPs is shown in Figure 2.

**Figure 3:**
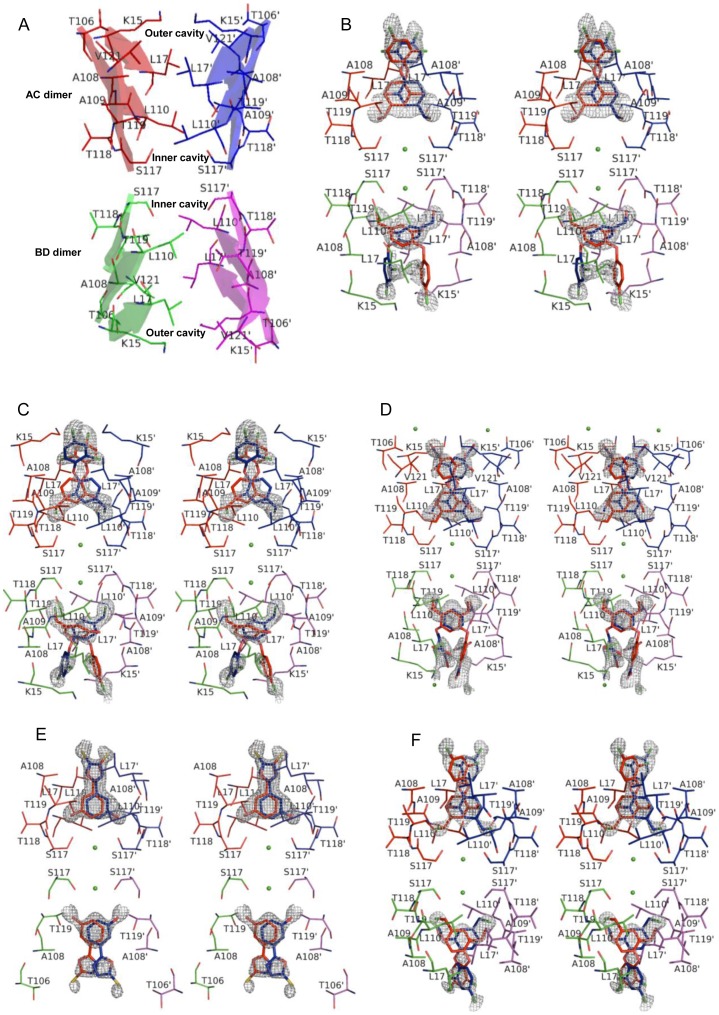
Crystal structures of TTR-ligand complexes. Two T4 (thyroxine) binding sites are shown with the bound ligand in each case. Interacting residues and the chemical structures of the bound ligands are shown as thin and thick sticks, respectively. The two T4 binding sites can be found at the interface of the AC and BD dimers. The portion of TTR dimers involved in making the T4 sites are shown as transparent cartoon and monomer A, B, C, and D are colored as red, green, blue and magenta, respectively. Stereo representation of all structures is shown and the TTR amino acid residues that contact ligands are shown as sticks and labelled in each case. The difference maps calculated for the ligand are contoured at σ = 3.0. Ligands binds in the two T4 binding sites, each ligand (red) generates a symmetric copy of itself (blue) which appears superimposed on it owing to its positioning on the twofold symmetric axis. Primed amino acids refer to symmetry related monomers of TTR. (A) Schematic of the two T4 binding sites without the ligand highlighting the residues forming the inner and outer binding cavities; (B) wtTTR-C2; (C) wtTTR-C3; (D) wtTTR-C6; (E) wtTTR-C1; (F) wtTTR-C7.

Ligands C2, C3 and C6 binds TTR in the ‘forward’ binding mode, where it’s polar substituents occupying the outer cavity allowing their ionic substituents to interact with the Lys15 and Lys15′ ε-NH3^+^ groups (Figure 3B, C and D). In contrast, ligands C1 and C7 bind in the ‘reverse’ binding mode, by extending their polar groups substituted aryl rings into the inner cavity to hydrogen bond with Ser117 and Ser117’ (Figure 3E and F).

### Binding and Interactions of wtTTR with C2

The compound C2 binds in forward mode by orienting its aldehyde and alcohol substituted aryl ring in the inner binding pocket (Figure 3B). Ligand C2 has better fit inside AC site than the BD site (Figure 3B). Type and the number of the interacting residues in two sites are also different (Figure 3B and Table 2). The thermal parameters of the ligand in AC and BD sites were 46.0 and 54.0, respectively. Interestingly, average B factors for the interacting residues from both the BD and AC sites were ∼22.0. Besides, making polar interactions with the amide nitrogen and carbonyl oxygen of Thr118-Thr118′ and Thr119-Thr119′, the oxygen atom of the aldehyde group also makes two hydrogen bonds with the side chain oxygen atoms, Oγ and Oγ^1^ of Ser117-Ser117′ and Thr119-Thr119′, respectively, in AC dimer. However, in second T4 site the same aldehyde oxygen atom make only single hydrogen bond with the hydroxyl oxygen (Oγ) of Ser117-Ser117′. Additionally, in BD site polar interactions with amide nitrogen of Leu110-Leu110′ and Thr118-Thr118′ are also observed (Figure 3B). The hydrophobic and van der Waals contacts are fewer in BD dimer as compared to the AC dimer (Table 2). They are contributed by Leu17, Ala108, Ala109, Leu117 and Thr119 in AC site whereas by Lys15, Leu17, Ala108, and Leu110 in BD site. As a consequence of changed conformation in BD site the ligand make several hydrophobic and van der Waals contacts with aryl ring in the outer binding pocket with Lys15-Lys15′ and Leu17-Leu17′ (Figure 3B). Side chains of Lys15-Lys15′ also make polar contacts with the substituted chlorine atom in the BD site only. In this complex two water molecules are also present between the Ser117 and Ser117′ in AC and BD dimer facilitating direct hydrogen bond with the side chain oxygen atom of Ser117 in all subunits. The negative cooperativity is further apparent by disordered electron density of the ligand in BD site (Figure 3B).

**Table 2:** Comparison of number of contacts made by each ligand with monomer A and B of wtTTR.

TTR residues	C1	C2	C3	C6	C7
Lys15A			10	2	
Leu17A	3	2	9	1	3
Thr106A				1	
Ala108A	2	6	6	5	6
Ala109A		2	8		4
Leu110A	6		6	2	6
Ser117A	6	10	7	10	7
Thr118A	2	4	3	4	4
Thr119A	3	5	3	4	3
Val121A				3	
Lys15B		5	4	7	
Leu17B		7	6	7	2
Thr106B	1				
Ala108B	2	2	3	2	5
Ala109B			4		1
Leu110B		4	8	3	5
Ser117B	1	5	7	5	8
Thr118B		2	3		4
Thr119B	4	3	2	1	3

### Binding and Interactions of wtTTR with C3

The compound C3 has substitution of a methyl halide group in place of aldehyde group which is present in C2 compound. Compound C3 adopt different conformations in two T4 binding sites, but unlike C2 it has identical interacting residues in both AC and BD dimers (Figure 3C). Nonetheless, the numbers of interaction in two sites are considerably different (Table 2). The chlorobenzene group of C3 exhibit electrostatic interactions with Lys15-Lys15′ in both AC and BD site albeit at a longer distance in BD site. In the outer binding pocket of AC site, residues Lys15 and Leu17 interact with the carbon atoms of the aryl ring. Ala108, Ala109, Leu110, Ser117 interact with carbon skeleton of methyl chloride substituted aryl ring. Same residues interact in BD site, but make fewer contacts as compared to AC site (Figure 3C). Presence of chlorine instead of aldehyde could make stronger polar interactions with the inner binding site by making electrostatic contacts with the side chain and main chain oxygen atoms of Ser117-Ser117′ and amide nitrogen of Thr118-Thr118′ in AC site (Figure 3C). As compared to BD site, several potent polar interactions with Thr119-Thr119′ and Ser117-Ser117′ in AC site further stabilize the ligand (Figure 3C). Unlike C2, outer binding pocket has amine moiety of Lys15-Lys15′ side chain mediated polar contacts with chlorine atom in both dimer interfaces (Figure 3C). Polar interactions mediated by the two ordered water molecules are observed with Ser117 and Ser117′ in this complex also. The thermal parameters of the ligand in AC and BD sites were 46.0 and 56.0, respectively. Interestingly, average B factors for the interacting residues from both the BD and AC sites were ∼24.0. The electron density of the linker region is only partially visible in AC site; it is completely disordered for the ligand in BD site, this again proves the intrinsic negative cooperativity of two ligand binding sites (Figure 3C).

### Binding and Interactions of wtTTR with C6

The ligand C6 is identical to C3 in basic architecture with halides being substituted with nitro or hydroxyl groups and hydroxyl group with a methoxy group (Figure 1). The thermal parameters of the ligand in AC and BD sites were 48.0 and 56.0, respectively. Average B factors for the interacting residues from both the BD and AC sites were almost similar i.e. ∼23.0. The ether and hydroxyl substituents of C6 extend into inner binding cavity (HBP3 and HBP3′) and its nitro substituted aryl rings extend into the outer cavity as shown in Figure 3D. The hydroxyl group of C6 in AC binding site creates a hydrogen bonding network that is mediated through ordered water molecule bridging the Ser117-Ser117′ side-chain hydroxyls of adjacent TTR subunits, whereas in the second T4 binding site Ser117 and Ser117′ form hydrogen bonding network with water molecule present between them without involving the ligand atoms (Figure 3D). Moreover, the distance of these water molecules differs in two binding sites; it is 2.9 Å and 2.84 Å in AC and BD sites respectively. In the AC site, hydroxyl group make several polar interactions with Ala108-Ala108′, Leu110-Leu110′, Ser117-Ser117′, Thr118-Thr118′, and Thr119-Thr119′. In contrast, the same group in BD site interacts with the amide nitrogen of Leu110-Leu110′ and Oγ of Ser117-Ser117′ (Figure 3D). Nitro group make hydrogen bonds with the ordered water molecules in both sites. However, in BD site nitro group of the second aryl ring make several polar interactions with Lys15-Lys15′ (Figure 3D). However, the lengths of these bonds are longer in BD site which imply that ligand in this site have less potent interactions (Figure 3D). The linker region is positioned in the hydrophobic environment of HBP2 and HBP2′ (Figure 3D). The density for the ligand in BD site is disordered and the position and conformations of the two aryl rings is substantially different from AC site indicating negative cooperative binding behavior (Figure 3D).

### Binding and Interactions of wtTTR with C1

The compound C1 binds in the reverse mode by orienting hydroxyl group into the inner cavity. Interestingly, unlike the previous studies the interactions, conformations and the binding positions in both sites were different. Interacting residues from both sites are shown in Figure 3E, which clearly demonstrate differences in the fit of the ligands in two sites and their interactions. The thermal parameters of the ligand in AC and BD sites were 54.0 and 64.0, respectively. However, the average B factors for the interacting residues from both sites were almost similar. As a consequence of the conformational difference, the ligand has fewer van der Waals interactions in BD site as compared to AC site (Table 2). Disordered electron density of the entire ligand in the BD site further indicates compromised interactions, which is a consequence of intrinsic negative cooperativity of two binding sites (Figure 3E).

As shown in Figure 3E, AC site has more polar interactions than in BD site. Thr119-Thr119’ in both T4 sites have a flip of rotamer compared to native TTR structure, allowing the formation of direct hydrogen bonds with the hydroxyl group of C1 but only in AC site. The hydroxyl oxygen atom of C1 make another hydrogen bond with the hydroxyl group of Ser117- Ser117′ in AC site (Figure 3E). Hydroxyl group of the ligand has numerous polar interactions with the inner binding pocket residues (Figure 3E). In BD site same oxygen of C1 makes a polar interaction with main chain nitrogen of Thr119-Thr119′, besides engaging Ser117-Ser117′ through a hydrogen bond. Although this bond has a slightly longer distance than in AC site (Figure 3E). Two water molecules are also present between the Ser117 and Ser117′ in AC and BD dimer facilitating direct hydrogen bond with the Oγ atom of Ser117 in all subunits.

### Binding and Interactions of wtTTR with C7

Compound C7 binds to TTR in the reverse binding mode, with the ether and methyl chloride substituted aryl ring oriented in the inner binding pocket to interact with Ser117-Ser117’ (Figure 3F). In the inner binding pocket of the BD dimer, the aryl ring is stacked between the hydrophobic side chains of Ala108, Ala108′, Leu110, Leu110′, Thr119, and Thr119′. The halide substituent occupies HBP3 in the inner cavity (Figure 3F). In AC site chlorine of methyl chloride group of the aryl ring make electrostatic interactions with both carbonyl oxygen and the Oγ of Ser117-Ser117′, amide nitrogen and carbonyl oxygen of Thr118-Thr118′ and amide nitrogen and Oγ1 of Thr119-Thr119′. However, in BD site this chlorine atom have electrostatic interactions with the same residues but at closer distances (Figure 3F). In both the AC and BD dimers, Ser117 and Ser117′ orient their hydroxyl groups towards the ligand, contributing to additional hydrogen bonding network with the chlorine atoms in AC and BD sites (Figure 3F). An ordered water molecule is located between the adjacent Ser117 residues of the AC and BD dimers facilitating hydrogen bonding interactions between the side chains oxygen of Ser117-Ser117′ and is not involved in any direct interactions with the ligand (Figure 3F). However, the side chain of Ala108-Ala108′, Ala109-Ala109′ and Leu110-Leu110′ in both sites contribute to additional van der Waals interactions with the ligand. However, the number of interactions observed in the BD dimer is fewer as compared to AC dimer (Table 2). The thermal parameters of the ligand in AC and BD sites were 51.0 and 63.0, respectively. However, the average B factors for the interacting residues from both the BD and AC sites were ∼20.0. In addition to this, distinction in the conformation, the number of polar interactions and the total number of interacting residues implies the involvement of negative cooperativity in the two T4 binding sites even though electron densities for ligands are well ordered in both sites.

### Comparative Analysis of All the Complexes of wt TTR

Out of the reported structures, ligand C2 (IC_50_ = 0.52 µM) and C3 (IC_50_ = 0.85 µM) have almost identical IC_50_ (ligand concentration at which there is 50% reduction in fibril formation) values and therefore are most potent in preventing tetramer dissociation under amyloidogenic conditions [14]. As shown in Figure 1 they also have almost similar chemical structures and therefore based on the type of functional group and its interactions with the T4 binding site residues it can be inferred that the presence of the hydroxyl is most critical in imparting inhibitory potential (Figure 3B&C). The carbon skeleton of ligand C6 and C7 is identical to C2 and C3 but they have entirely different functional group substitutions. As evident from the Table 2 and Figure 3, the chemical difference between the substituent groups greatly affects their capabilities to form polar interactions with HBP3 residues. Similarly, in C6 presence of nitro groups in the place of the halogens in the C2, C3, and C7 has greatly affected its interactions and position inside the HBP1 leading to reduced interactions with side chain of the Lys15 as compared to the halogens. This is also reflected by higher IC_50_ values of the C6 as reported in the previous study [14]. Out of the reported ligands, C1 has intermediate IC_50_ value. It has oxadiazol ring with mercapto group in place of the second aryl ring. This modification renders it with lesser hydrophobic interaction with TTR, nevertheless presence of hydroxyl group in the HBP3 bestow it with better interactional network (Figure 3E). On the other hand, presence of pentameric ring instead of aryl ring provides better fit inside outer cavity. Based on this structural analysis it can be inferred that the balance of both hydrophobic and polar groups on biphenyl skeleton is very critical in determining their efficacies to inhibit the tetramer dissociation under amyloidogenic conditions.

**Figure 4:**
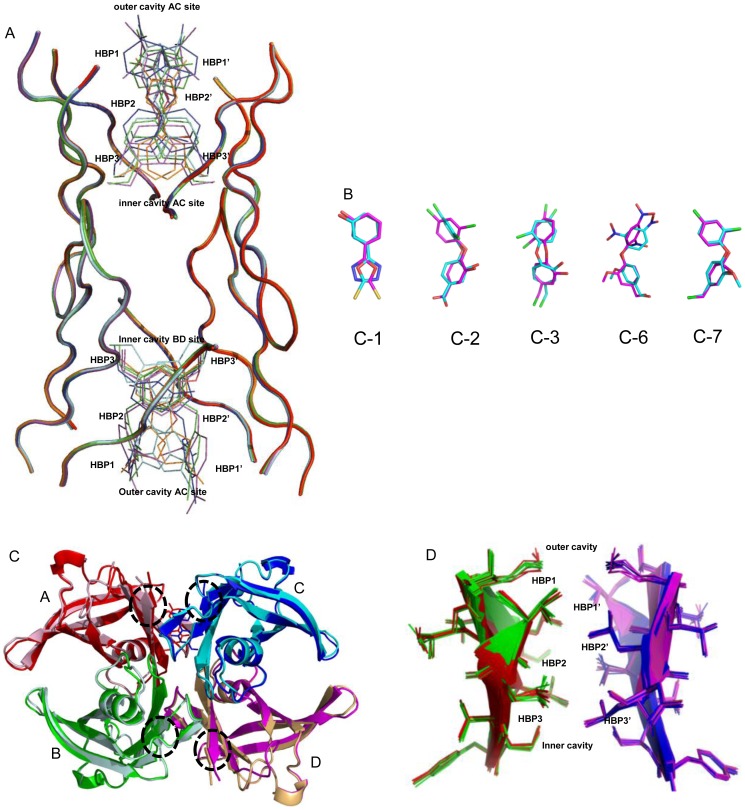
Comparison of different binding sites found in TTR-ligand crystal structures. (A) Superposition of the *apo* and *holo* forms of the wtTTR. Only backbone of the amino acids comprising the ligand binding site of AC and BD dimers along with bound ligands (thin sticks) are shown, *apo* wtTTR (black), wtTTR-C1 (red), wtTTR-C2 (green), wtTTR-C3 (blue), wtTTR-C6 (magenta), and wtTTR-C7 (cyan). (B) Superposition of the ligand molecules found in the AC (magenta) and BD (cyan) binding sites of the wtTTR:C1, wtTTR:C2, wtTTR:C3, wtTTR:C6, and wtTTR:C7 crystal structures. (C) Superimposition of the entire molecule of the wtTTR in *apo* form (monomers A, B, C and D are shown in pink, light green, cyan and light orange colors, respectively) and *holo* form (monomers A, B, C and D are shown as red, green, blue and magenta colors, respectively) is shown along with bound ligand in AC site only. Structural changes in comparison to the *apo* form are marked with the circles. (D) Superimposition of the BD dimer on the AC dimer from the all 5 complexes of wtTTR with ligands (only T4 binding site residues along with secondary structure is shown). Monomers A, B, C and D are shown in red, green, blue and magenta colors, respectively.

In all the complexes, the electron density of the aryl ring and linker atoms of the ligand are well ordered in the AC site and only partially ordered in the BD site. In addition to this orientations and the positions of the ligands in BD site were also different, indicating the outward movement of the ligands as a consequence of negative cooperativity between two sites. The ligands in BD site have high atomic B-factor values and thus poorly defined electron density. Polar and van der Waals interactions between residues underlying the inner cavity of BD site and atoms of the ligands are at significant distances as compared to those in AC site. Moreover, the bridging hydrogen bond network mediated by an ordered water molecule between hydroxyl of Ser117 and the chemical entities of the diverse ligand in BD site are at farther distances. This implies that the ligand-protein contacts at the entrance of the TTR binding channels are less robust in BD site as compared to the AC site. Except ligand C1, in all remaining structures, the aryl rings are non-planar with respect to each other, adopting out-of-plane orientations (Figure 4A).

**Figure 5:**
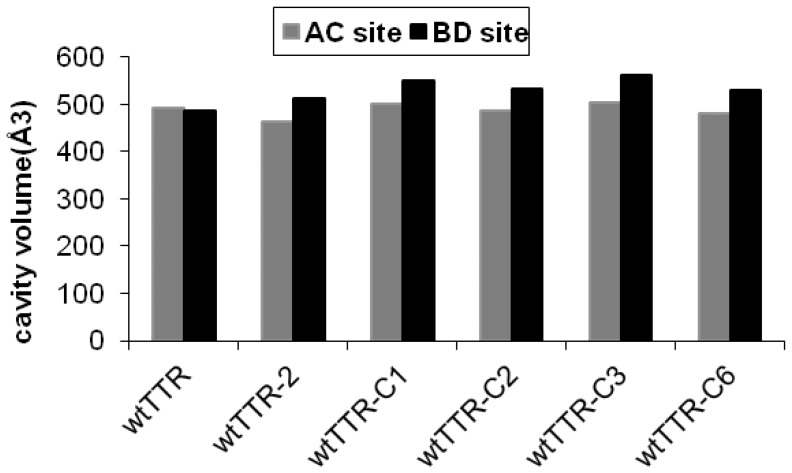
Comparative analysis of ligand binding cavity size. *Apo* and *holo* wtTTR binding site dimensions were calculated using the CastP server [31]. The entire molecule including the water molecules were used for cavity volume calculations.

To elucidate the local changes in the halogen binding pockets as a consequence of ligand binding, all ligand bound structures were compared with each other and the *apo* form of TTR (Figure 4 A). The overall structures of wtTTR in complex with these five ligands were kept unchanged upon complexation, as evidenced by the low (<0.5 Å) deviation in Cα distances between the aligned monomers (Figure 4A). Superimposition of all ligands bound in AC site with the one in BD site indicated major conformational distinctions between them (Figure 4B). Entire tetramer of the wtTTR in *holo* and *apo* form was superimposed to compare the conformational changes outside the ligand binding cavity. As is evident from the Figure 4C, there are some structural rearrangements at the opening of the ligand binding site. These changes show that in comparison to the *holo* form, A and C monomers are slightly pulled towards each other whereas B and D monomers are slightly pushed apart. These structural changes might be representing the final state of the ligand binding event, which leads to better interactions in AC site as compared to BD (Table 2). AC and BD sites of the *apo* and *holo* TTR were superimposed to see the conformational differences in two binding sites, which are illustrated in Figure 4D. In *holo* forms no residues other than Lys15, Ser117 and Val121 have differences in the side chain orientation. However, in comparison to the *apo* form the secondary structural element of *holo* form are slightly pulled towards each other in AC site whereas in BD site they are slightly pushed apart (Figure 4C). The B factors for the A and B chain in the *holo* forms on an average were ∼23 and ∼20, respectively. On the other hand the B factors of the A and B chains of the *apo* form on an average were 16 and 18, respectively. Thus, the B factors for the both chains of *apo* form were slightly lesser than those of the *holo* forms. A comparative analysis of ΔG values of the non crystallographic dimer AC (ΔG^diss^ ∼10.2 kcal/mol) and BD (ΔG^diss^ ∼6.3 kcal/mol) of TTR complexes indicated the AC dimer to be the primary site for ligand binding in all the complexes. The comparison of cavity volume in *holo* forms of TTR indicated compact AC site as compared to the BD, whereas the cavity volumes of two sites in *apo* form showed no considerable difference (Figure 5). This analysis further confirms that, the A and C monomers come slightly closer whereas the B and D monomers are pushed apart as a consequence of the favored interactions in AC site upon ligand binding (Figure 4C). Alterations in the network of interactions between Lys15, Ser117, Thr118 and Thr119 and the functional group of the ligands and the biphenyl core interactions with the cluster of residues including Thr106, Ala108, Ala109, and Leu110 at the dimer interface influence the binding affinity of second ligand. Additionally, network of water molecules around the region comprising of residues 105–115 is different in all the complexes, indicating that displacement of water molecules during the ligand binding might be influencing the interactions of the ligand with T4 binding site. Disruption of the interactions of this stretch has been shown to be most important for the amyloid fibril formation by the monomers in the dissociated state. As shown in Figure 5 the larger cavity volumes, differences in ligand interactions in two sites, consistently electron density of all the ligands and their higher thermal factors in BD site provide a persuasive evidence for the proposed mechanism underlying the negative cooperative binding in two T4 sites.

## Discussion

Evaluation of the overall protein movements in TTR tetramer induced by ligand binding showed no significant changes in the hormone binding site. The RMSD values were within 0.23 Å. However, the side chains rotations of Lys15-Lys15′ and Ser117-Ser117′ (present at the closing and opening of the T4 binding pockets, respectively) in all complexes create different conformations. These changes in side chain rotations reflect the adjustments in outermost and innermost hormone binding sites for entry of the ligand and exit of the water molecules in each case. Further, these subtle changes facilitate better fit of the ligand in AC site and play a significant role in imparting kinetic stability under amyloidogenic/denaturing conditions.

Analysis of different structures of TTR have revealed the presence of 4 & 6 conserved water molecules in the buried core of A and B chain, respectively. Among these conserved water molecules, one in each monomer seems to play an important role in the ligand binding with the protein. Comparative analysis of unliganded TTR and TTR-ligand complexes reveal that in the unliganded structures the conserved water molecule forms hydrogen bond with the side-chains of two important residues, Ser-117 and Thr-119 of the thyroxine binding pocket. The Ser-Thr bound conserved water molecule seems to migrate when the ligand molecule enters in the pocket and forms complex with the protein. Again, during migration of that water molecule, side chains of the respective Ser-117 and Thr-119 adopt a trans like conformation and stereo-chemically assist the ligand to occupy the specific binding pocket of TTR. However, the positional invariance of two water molecules between the two T4 sites for enhancing the water mediated polar contacts with ligands in each case are interesting.

Comparison of the ligand molecules in the AC site with those in the BD site of the wtTTR-ligand crystal complexes reveals that the ligand molecule occupying the BD site is positioned differently in comparison to the ligand molecule in the AC site. The analysis of hormone binding pocket dimensions in the crystal structure of wtTTR *apo* and *holo* forms show that the binding site dimensions in the *holo* forms of wtTTR are slightly dissimilar to those found in *apo* wtTTR. Moreover, a detailed inspection of the binding sites in all complexes demonstrated that the interactions in BD site are minor in number and at longer distances. Therefore, these snapshots either represent a stage wherein one ligand is moving out of the T4 site or there is a preference of one site to be the primary site for ligand binding. Our findings are corroborated by the observation that occupancy of only 1 of the 2 possible sites was sufficient to stabilize the entire tetramer and prevent amyloidogenesis [15]. Additionally, it has been shown that stoichiometries of ligand with TTR are always less than 2 under physiological conditions, implying that both sites are not occupied simultaneously [17].

A large number of ligands have been studied for their interaction with TTR and their aggregation inhibitory activity. Some of the reported ligands display negative cooperativity in the interaction with wtTTR, usually with the *K*_d1_ in the nM and *K*_d2_ in µM range [12], [13], [14]. However, so far none of the studies provide structural basis of this behaviour. Another recurrent feature of ligand binding to TTR is, its ability of interaction with all these compounds with minor or no effect in global protein structure, regardless of crystallization conditions, space group and ligand orientation. Comparison between the present TTR complexes and previously reported structures [18], [19], [20], [21], [22] show considerable differences in the interactions and orientation of ligands within one of the T4 binding sites with implications for a negatively cooperative binding process.

As evidenced by structural analyses reported here, all five ligands bind to the two sites of wtTTR in a negatively cooperative manner and display distinct interactions in two T4 sites. Differences could be observed in the ligand positions in the wtTTR channel. The substitution of 3-hydroxyl in triclosan with polar groups such as aldehyde (as in the case of C2) increases the propensity for polar interactions with either Lys15-Lys15′ or Ser117-Ser117′. In other words, the presence of negatively charged groups influences their binding to wtTTR and thereby increasing their affinity for hormone binding channel. The negative cooperativity as evidenced in all these complexes imply that a general mechanism can be deciphered, which involves robust interactions in one site upon binding of a ligand leading to altered interactions between TTR subunits which consequently compromises the binding of the second ligand molecule. This mechanism ultimately leads to slight pulling apart of the other crystallographic dimer. The following opening of the first, already occupied site, changes in the interactions upon ligand binding in the second site is smaller because of the bridging interactions have already formed by the first ligand. The binding event shows that a ligand binding at first binding site causes the collapse of the interactional network in the second binding site leading to its slight expansion and thus compromised interactions with the second ligand. It shows that the tetramer’s central channel has intrinsic asymmetric control of two binding sites.

## Experimental Methods

### Preparation of Ligands

The compound C1 was purchased from Chemical Diversity Labs Inc, San Diego, CA. All biphenyl Ether (BPE) substituted compounds were synthesized as reported previously [14]. Stock solutions (10 mM) of the compounds were prepared in DMSO. All compounds diluted from the stock solution were solubilised in phosphate buffer prior to soaking experiments.

### Expression and Purification of wt-TTR

TTR cloned in pMMHa vector was a kind gift of Dr. P. Raghu from National Institute of Nutrition, Hyderabad, India. Recombinant wtTTR were expressed and purified as described previously [23]. Protein concentrations were determined by measuring absorption using the absorption extinction coefficient of 7.76×10^4^ M^−1^ cm^−1^ at 280 nm. Protein purity was assessed by SDS-PAGE, and its mass was confirmed by electron spray ionization mass spectrometry (ESI-MS).

### Crystallization

All TTR-ligand complexes were prepared from crystals soaked with a tenfold molar excess of ligand for 2 days to ensure saturation of both binding sites without affecting the diffraction quality of the crystals. For crystallization, TTR (10–15 mg/ml) in 10 mM phosphate, 100 mM KCl, and 1 mM EDTA, pH 7.4 was equilibrated against 2 M ammonium sulfate in 4 µL hanging drops. Crystals appeared after 4 weeks and had well-defined geometric forms.

### X-ray Data Collection, Structure Determination and Refinement

A Mar345dtb image plate coupled to a Rigaku RU300 rotating anode X-ray generator, operating at 5 kW was used for data collection of all five complex structures and images were collected with an oscillation of 1° per image. A single crystal was placed in 33% glycerol containing mother liquor as a cryoprotectant and cooled to 120 Kelvin for diffraction experiments. Data reduction for all data sets were performed using MOSFLM [24] and SCALA [25]. Crystals of all TTR-ligand complex structures are isomorphous with the *apo* crystal form with the space group P2_1_2_1_2 with two monomers in the asymmetric unit (Table 1). From the X-ray structure of *apo wt*TTR (PDB ID: 1DVQ [19]), chains A and B together, but without water molecules, ware used as an initial model for molecular replacement using the program PHASER [26]. The initial protein atomic model was subjected to rigid body refinement using chains A and B as independent rigid body domains in REFMAC [27]. The model was improved through alternated cycles of real space refinement using COOT [28], maximum likelihood minimization using restrained refinement protocols in REFMAC5 [27]. Difference Fourier maps revealed distinct electron density for the ligand in each complex. The ligands were manually fitted in the unbiased omit maps which were calculated using the program CNS [29]. Each of the ligand lies on the two fold symmetry on the crystallographic Z axis. For this reason, ligand molecules were initially refined with occupancy of 0.5. Water molecules were identified using find water module of COOT [28] in the last steps of refinement. Online server MolProbity [30] was used to check the quality of the refined structures. All the figures were prepared using PyMOL (http://www.pymol.org).

### Accession Codes

The coordinates and the structure factors have been deposited in the protein data bank (http://www.pdb.org) under the accession codes 4ABQ, 4ABU, 4ABV, 4ABW, 4AC2 and 4ANK.
